# Experiences of stigma among caregivers of children with disabilities in Freetown

**DOI:** 10.1038/s41598-025-07034-1

**Published:** 2025-06-20

**Authors:** Hanna Luetke Lanfer, Elizabeth Anderson, Fatmata Bah, Sarah Krawiec, Constanze Rossmann, Anna Vines

**Affiliations:** 1 School of Public Health, Bielefeld University, Universitaetsstrasse 25, 33615 Bielefeld, Germany; 2 Enable the Children, World Hope International, Freetown, Sierra Leone; 3 Freetown, Sierra Leone; 4 Department of Media and Communication, LMU Munich, Munich, Germany

**Keywords:** Stigma by association, Affiliate stigma, Disability, Caregivers, Sierra Leone, Social exclusion, Children living with disabilities, Human behaviour, Public health, Patient education

## Abstract

Stigma surrounding disability continues to shape the lives of affected individuals and their families across diverse contexts. Studies indicate that caregivers of children living with disabilities (CLWDs) face stigma through their close association with the CLWD and the internalization of these beliefs. In Sierra Leone, experiences of stigma among caregivers may be intensified by beliefs that link disability to spiritual or moral failings of the CLWD’s family and extend blame for the child’s condition to the caregiver. This study explores how caregivers experience and respond to these intersecting forms of stigma and the social support they encounter or desire. We conducted six focus group discussions with 37 caregivers of CLWDs in Freetown, Sierra Leone, recruited through hospitals and a non-governmental organization providing therapeutic services. Discussions were guided by a semi-structured interview protocol and analyzed using qualitative content analysis. Caregivers reported experiencing stigma through labeling, avoidance, societal blame, and coercion into traditional practices. Stigma was deeply rooted in cultural narratives that attribute disability to supernatural causes, often extending blame to caregivers. Participants employed three overarching strategies to navigate stigma: withdrawing, coping (e.g., spiritual reframing, focusing on positive interactions), and resisting (e.g., challenging superstitious beliefs, advocating for inclusion). Despite some positive experiences of empathy and social inclusion, caregivers expressed a strong need for more practical support, community awareness, and holistic interventions to reduce stigma and foster understanding. This study sheds light on how intersecting forms of stigma—stigma by association, attributions of blame, and internalized stigma—shape the experiences of caregivers in Sierra Leone. While participants demonstrated resilience and adaptive strategies, the dual stigma of association and societal blame underscores the urgent need for inclusive policies and community-based interventions. Amplifying the voices of caregivers and addressing their needs holistically are essential steps toward reducing stigma and creating supportive environments. This study contributes to the broader discourse on caregiving and stigma, offering insights for research and interventions in similar cultural contexts.

**Supplementary Information:**

The online version contains supplementary material available at 10.1038/s41598-025-07034-1.

## Introduction

Stigma refers to a distinguishing “mark” that differentiates individuals based on specific attributes, characteristics, conditions, or behaviors, often framing them as deviations from societal norms^1–3^. These marks, however, are not inherently positive or negative. Instead, through social processes, they acquire meaning, leading to the attribution of value or disvalue depending on the cultural and relational context^3^. Stigma thus operates as a socially constructed phenomenon, simultaneously affirming the “normalcy” of some while marginalizing others^1,3^. A trait that stigmatizes one person hence affirms the normalcy of another, illustrating that stigma is constructed within social and relational contexts^1,3^. Stigma operates on multiple levels: through societal structures, interpersonal interactions, and internalized beliefs, profoundly shaping the experiences, opportunities, and well-being of those affected^4–7^.

Disability—whether visible or invisible—has historically been framed as a mark. Disability has often been constructed as a deviation from societal norms and stigmatized across different societies and historical periods^8,9^ as societal expectations of able-bodied norms create frameworks that casts persons living with disabilities (PLWDs) as deviant or burdensome^10,11^. While research on stigma has traditionally focused on the direct experiences of stigmatized individuals, such as PLWDs^12^, stigma also extends to those associated with the stigmatized—families, caregivers, and close connections—leading to what is termed stigma by association^13–15^. Caregivers of persons with disabilities often face forms of social marginalization similar to those experienced by PLWDs, leading to affiliate stigma, the internalization of negative beliefs about the associated stigma^15,16^. In certain sociocultural contexts, however, this may extend beyond association, as caregivers can also be blamed or held responsible for the condition, exposing them to additional forms of responsibility or moral stigma^12,17^.

This phenomenon is particularly pronounced in cultural contexts where disability is imbued with additional layers of meaning. In Sierra Leone, disabilities—especially those by birth or acquired without an accident—have been interpreted through a cultural lens that associates them with curses, sin, or moral failings within the family^10,17,18^. This belief system then stigmatizes the individual with a disability but also extends to parents, who may be held responsible or blamed for the condition^12,17,19^. Studies from other settings with similar cultural frameworks have shown that parents and other family caregivers deal with the practical challenges of caring for a person with a disability and different, intersecting forms of stigma, ranging from stigma by association with the stigmatized person, culturally informed attributions of responsibility for the condition and the internalization of stigmatization^15,20^.

Research on the needs and experiences of stigma of people living with disabilities in Sierra Leone remains underexplored, even more so the experiences of their caregivers. In our study, we aim to respond to this gap in the literature by examining how caregivers of children living with disabilities (CLWD) in Sierra Leone encounter and respond to intersecting forms of stigma linked to their children’s conditions. By focusing on the caregivers’ perspectives, this research seeks to shed light on how social and cultural beliefs around disability shape compounded stigma experiences, including those grounded in stigma by association, attributions of blame or culpability that position the caregiver as the source of the condition, and internalization of the stigma. Understanding these experiences is crucial for developing interventions that address not only the needs of individuals with disabilities but also those of the families who support them.

## Background

### Conceptual overview of stigma

The term ‘stigma’ originates from Ancient Greece, where it referred to physical marks branded onto individuals, such as slaves, to signify their inferior social status and to mark them as objects of exclusion from societal privileges^1^. Over time, the concept evolved beyond physical markings to encompass a broader social and symbolic process of discrediting individuals or groups based on attributes or conditions perceived as deviating from societal norms^21,22^. Stigma manifests in social interactions, hence when society identifies a difference, labels this as negative and differentiates between those with and without the mark^15,16,22^. Addison^7^ therefore suggests conceptualizing stigma as a verb to emphasize that it is enacted between people and gains its meaning relationally. Stigma functions as a mechanism of social control by discouraging nonconformity with the norm (e.g., ostracizing individuals with unconventional lifestyles), upholding the values of a given society (e.g., emphasizing economic productivity and performance as ideals, often marginalizing those unable to meet these standards), and facilitating social cohesion between those who adhere to the dominant norm (e.g., fostering in-group solidarity by excluding or marginalizing those with disabilities)^1,22–24^.

Stigma is not a static label but a dynamic process rooted in historical, socioeconomic and cultural contexts^3^. How and whether a certain attribute is perceived, (dis)credited, and constructed as stigma, is highly context-dependent and contingent on cultural values and societal power dynamics that can change^3,24^. The attributes themselves are not inherently positive or negative; rather, they are assigned value through social processes. Historically, disability has been framed as a personal tragedy, moral failing, or sign of spiritual disharmony, justifying stigmatization and social exclusion^25^. The medical model, which dominated the nineteenth and twentieth centuries, reinforced this deficit-based view by framing disability as problematic, requiring treatment or cure and thereby further marginalizing PLWDs^25,26^. Over time, the social model of disability emerged, reframing disability as the result of societal and environmental barriers, such as inaccessible infrastructure and discriminatory practices, rather than individual impairments^26,27^. This reframing positions disability as arising from the failure of societies to accommodate diverse needs, rather than from inherent limitations within individuals. Although the World Health Organization’s definition of disability as well as international policies reflect this shift, institutionalization, exclusion, and dependency-based frameworks remain prevalent and continue to perpetuate the marginalization of PLWDs^28,29^. Moreover, in many capitalistic, achievement-oriented societies that prioritize able-bodied norms, disability continues to be perceived as a marker of difference or inferiority within societies, as success and economic contribution are often narrowly defined through productivity, independence, and physical or cognitive performance^7^.

### Types and levels of stigma

Goffman^1^ identified three primary types of stigma: (1) visible stigma, (2) acquired stigma, and (3) tribal stigma. (1) Visible stigmas are those immediately apparent to others, such as physical disabilities or disfigurements. These stigmas often provoke immediate reactions and judgments, as they disrupt societal expectations of ‘normalcy’ in appearance or function^30^. For example, individuals with conditions like Down syndrome, cerebral palsy or physical impairment needing a wheelchair may face gazes as their visible mark draws attention and reinforces societal discomfort with physical or cognitive differences^31–33^. (2) Acquired stigmas are associated with behaviors, conditions, or life circumstances that develop over time, such as mental illness, addiction, or chronic illness, which may result in disability. These stigmas are often intertwined with moral judgments, as they are perceived to be within an individual’s control, whether accurately or not^22,34^. (3) Tribal stigmas are tied to certain group affiliations, such as race, ethnicity, religion, or cultural identity, some of which are visible whereas others are not^35,36^. Tribal stigma is often inherited or culturally transmitted^1^. In contexts like Sierra Leone, disability is sometimes intertwined with cultural and spiritual beliefs, where individuals with congenital conditions are stigmatized as bearing ancestral curses or signs of witchcraft^6,25^. These types of stigma are not mutually exclusive and often intersect in complex ways^37^. For example, an individual with a visible physical disability who is also a member of a racially marginalized group may experience layered or compounded stigmatization that amplifies their social exclusion^3,38^.

Furthermore, stigma operates at multiple, interconnected levels—public, individual, and structural—each influencing and reinforcing the others^15^. Public stigma, also referred to as interpersonal or social stigma, emerges through collective attitudes, behaviors, and social interactions and serves as the foundation for other forms of stigma^39,40^. It reflects societal norms that define certain traits, conditions, or behaviors as deviant, shaping how individuals perceive and react to groups that do not possess the desired characteristics. For example, public stigma continues to be evident in derogatory language for disabilities, such as cripple or crazy^41^. These public perceptions and reactions have shown to fuel individual-level stigma, where negative stereotypes become internalized within the person not conforming with the dominant norm^42^. Self-stigma due to experiencing public stigma has been associated with psychological distress, anxiety, stress and lowered quality of life experience^43,44^. Structural stigma, where discrimination is embedded within societal policies and institutions, manifests in inaccessible public spaces, limited disability-specific healthcare, and inadequate educational provisions for CLWDs^45,46^. While these levels are conceptually distinct, they frequently intersect, creating a compounding effect on the lives of those who are stigmatized. The intersection of structural and public stigma, for instance, can amplify the exclusion of individuals with disabilities, as public attitudes shape institutional policies that fail to address their needs adequately^46–48^.

### Stigma by association and affiliate stigma

Stigma is not limited to individuals directly marked by a discrediting trait; it extends to those associated with stigmatized persons, such as caregivers^1^. This phenomenon, referred to as stigma by association or courtesy stigma, highlights how societal perceptions of deviance can ‘spill over’ to those in close relationships with stigmatized individuals, such as family ties, friendships, or even professional associations^1,16,49^. Stigma by association reflects the external, relational dynamics of being connected to a stigmatized individual, manifesting through public blame, judgment, and social exclusion. Birenbaum^16^ describes those associated with a stigmatized person as “’normal’ yet ‘different’. Their normality is obvious in their performance of conventional social roles; their differentness is occasionally manifested by their association with the stigmatized during encounters with normal” (p. 196). Thus, a central distinction between primary stigma and stigma by association lies in its visibility. For individuals with stigmatized attributes, visibility often is a defining feature of their stigma, whether immediately apparent or revealed over time. For stigma by association, however, the visibility of the stigma is contingent upon their proximity to the stigmatized person^13,15,50^. For instance, when caregivers are with their child who has a disability, they may be subject to public stigma, including intrusive questions or overt discrimination^20^. Yet, in contexts where the child is absent, caregivers can, to some extent, escape the immediate effects of stigma by association and decide whether to disclose or not their association with the stigmatized person^16,49^. This situational fluidity contrasts with the experiences of the stigmatized individual, whose attributes remain largely constant regardless of context^16,30^. However, even in the absence of the child, caregivers may carry the internalized effects of the stigma by association^16,51^. This process is referred to as affiliate stigma, the internal psychological impact on caregivers as they internalize societal prejudices and stereotypes about the stigmatized individual independent of public scrutiny, leading to feelings of guilt, shame and stress as multiple studies show (e.g.^30,52–56^).

Another form of stigma may emerge in contexts where cultural or religious beliefs, such as curses or parental wrongdoing, are associated with a child’s condition. Caregivers may not only be associated with the child’s condition but also be viewed as responsible for the disability itself. These forms of moral attribution can position the caregiver as the source of the condition, producing a form of acquired or primary stigma and, thereby, intensifying feelings of shame and blame among caregivers^11,20,57^.

### Disability in Sierra Leone

Disability in Sierra Leone encompasses a broad range of conditions, including physical impairments (e.g., amputations from war injuries or mobility limitations from accidents exacerbated by unsafe working and living conditions common in sub-Saharan Africa), sensory disabilities (e.g., blindness and deafness), and intellectual and developmental disabilities (e.g., cerebral palsy, Down syndrome, and autism)^58^. Moreover, while chronic illnesses, such as diabetes or sickle cell disease, are not disabilities in themselves, they can result in disabling conditions over time when untreated or poorly managed^59^. The exact prevalence of disability in Sierra Leone remains unclear due to unreliable data collection and a lack of comprehensive surveys. Reports suggest that the needs of PLWDs are “grossly underestimated,” highlighting significant gaps in understanding and addressing disability in Sierra Leone^58^ (p. 16). This mirrors trends across sub-Saharan Africa, where a high proportion of the population experiences disability and chronic illnesses, often without adequate access to healthcare or support systems^60^. These conditions are further compounded by the socio-political and cultural environment, which influences how disability is understood and experienced^38,61^.

Disability in Sierra Leone is also shaped by a complex interplay of historical, cultural, and socio-political factors. The legacy of the civil war (1991–2002) influenced how different types and forms of disability are perceived and experienced, as Berghs and Dos Santos-Zingale^18^ concluded from their anthropological work. During and after the conflict, individuals with war-related disabilities, such as amputations, categorized themselves as ‘victims’ to access resources and support whereas the label ‘disabled’ was often associated with social devaluation and dependency^18,62^. The ‘victim label’ was seen as more socially acceptable and aligned with reparative justice frameworks for war amputees^63^. In contrast, individuals with other disabilities have not benefited from similar levels of support or say in defining their public image (ibid). Moreover, especially congenital or early-onset conditions, like cerebral palsy that occur before, during or shortly after birth, continue to be rooted in traditional and religious beliefs, such as viewing disability as a curse or divine punishment^10,64^. These beliefs hence also extend the condition to the family, especially parents, of the CLWD who might be perceived as bearing responsibility for the condition^65^. In this context, where certain disabilities are attributed to supernatural or moral causes, caregivers may be socially marked through their association with the child, held responsible for causing or contributing to the condition, and internalize the affiliate stigma. These different forms of stigma may overlap and reinforce one another, shaping how stigma is experienced and responded to. To our knowledge, the intersecting forms of stigma experienced by caregivers of CLWDs in Sierra Leone have not been explored in Sierra Leone. To address these underexplored dynamics, this study was guided by the following research questions:

- RQ1: In what ways do caregivers of CLWDs in Sierra Leone experience stigma?
- RQ2: In what ways do caregivers of CLWDs in Sierra Leone respond to stigma?
- RQ3: What social support do caregivers of CLWDs in Sierra Leone experience and wish for?

## Methods

### Study design

To explore the intersecting experiences of stigma experienced by caregivers with CLWDs in Sierra Leone, an exploratory qualitative study was conducted using focus group discussions. The study sought to examine how caregivers experience, interpret, and respond to different forms of stigma within their everyday lives. Focus groups were chosen to encourage collective reflection on shared experiences and divergences across caregiving contexts. Logistical support, including facilitation of participant recruitment and coverage of field costs (e.g., refreshments, contract of a local researcher), was provided by the local non-governmental organization (NGO), World Hope International’s Enable the Children (ETC) program, which offers therapeutic and psychosocial services to children with disabilities and their families. All other authors volunteered their time without dedicated funding.

### Recruitment

Given the role stigma plays in shaping the experiences of caregivers, this study specifically sought to engage those who had not yet been supported by ETC, as their perspectives could provide unique insights into the challenges faced before accessing formal support systems. To reach this group, the following recruitment approach was taken. ETC collaborates with several hospitals across Freetown, where their presence ensures a connection to CLWDs and their caregivers when they present themselves for medical care. Healthcare staff at these hospitals, upon identifying CLWDs whose conditions might benefit from ETC’s therapy services, provide referrals to the organization. As part of this process, caregivers who received a recommendation from hospital staff were then contacted by ETC personnel. During these initial interactions, caregivers were informed about the research project and invited to participate. To mitigate any perceived pressure or conflict of interest due to ETC’s dual role in recruitment and service provision all caregivers were informed, verbally and through an information sheet, that their decision to participate or not would have no impact on their eligibility for ETC’s therapy or support services.

### Data collection

After establishing contact, all participants received an information sheet outlining the project’s objectives, data processing procedures, and measures to ensure data protection. Recognizing the low literacy rates among the population, the consent form was read and explained orally to each participant to ensure comprehensive understanding. Participants were given the opportunity to ask questions about the study and, if desired, discuss the consent form with a literate family member, friend, or trusted individual. Consent was obtained through a thumbprint or signature, ensuring that participants could provide informed consent regardless of their literacy level.

We conducted six focus group discussions with five to seven participants in different hospitals across Freetown in the rooms used by the NGO from December 2021 to April 2022 (see Table 1). The focus group discussions were held in Krio, the most widely spoken lingua franca in Sierra Leone and commonly spoken across different ethnic groups. The group discussions were conducted by a local researcher (FB) who was contracted for this research project and guided by a semi-structured interview guide. Questions focused on sociodemographic data of the participants and the CLWD they took care of, experiences of stigma, response to stigma, and social support needs (see Supplementary material for an overview of the question guide).

**Table 1:** Overview of focus group discussions.

Number of focus group	Date	Location	Participants per group
FGD 1	Dec 4, 2021	Ola During Children’s Hospital, Freetown	6
FGD 2	Dec 4, 2021	Ola During Children’s Hospital, Freetown	5
FGD 3	Apr 2, 2022	Aberdeen Women’s Hospital, Freetown	7
FGD 4	Apr 2, 2022	Aberdeen Women’s Hospital, Freetown	7
FGD 5	May 14, 2022	Ola During Children’s Hospital, Freetown	7
FGD 6	May 14, 2022	Ola During Children’s Hospital, Freetown	5

Discussions were audio-recorded and lasted between 50 and 60 min. The translation to English and the transcription process occurred simultaneously. To allow for transcript accuracy and avoid the loss of meaning in the translation process, the focus groups were transcribed and translated by the researcher who conducted the group discussions. Transcripts were later discussed with the rest of the research team, who are all familiar with the sociocultural context of Sierra Leone and speak Krio. In the case of a metaphoric expression that does not have an equivalent in English or cultural differences which need a context explanation, a note with an explanation of the meaning of the expression was made in the transcript. Any personal data of the participants was pseudonymized, the audio recordings were deleted after transcripts were finalized.

### Data analysis

Data were analysed using qualitative content analysis, supported by the software MAXQDA 2024 (VERBI GmbH). The initial coding frame was developed by HLL using the first two focus group discussion transcripts, incorporating both predefined themes and inductively derived subcategories. SK then applied this preliminary coding frame to the same two transcripts. Areas of inconsistency or ambiguity were discussed, and the coding frame was refined accordingly. The remaining four transcripts were coded independently by both researchers. After completion, the coded transcripts were compared and all discrepancies were reviewed. Final coding decisions were made through discussion until full consensus was reached.

### Statement of positionality

This research was conducted by an all-female team with diverse cultural and professional backgrounds, combining insider and outsider perspectives on the Sierra Leonean context. HLL, a foreign academic with extensive research and work experience in Sierra Leone, lived in the country for several years and speaks Krio. She oversaw the study. The study was initiated and designed by EB and AV, who are expatriate physiotherapists, based in Sierra Leone for several years, with AV having lived and worked in the country for over a decade. Both have long-term engagement in therapy services for children with disabilities and are fluent in Krio, with in-depth understanding of local customs and family dynamics. A local researcher (FB) supported recruitment and conducted all focus group discussions, contributing essential cultural and linguistic insight throughout data collection. SK and CR, both Germany-based academics, contributed to the data analysis and conceptual framing of the study. The research team was conscious of how their own backgrounds, particularly as individuals without disabilities, may have influenced their perspectives on stigma and caregiving experiences. Additionally, we acknowledge that our gender may have shaped participant interactions, particularly in relation to societal norms around caregiving and discussing sensitive topics. By integrating the expertise of local practitioners and researchers with international academic perspectives, the study sought to balance critical analysis with cultural sensitivity, ensuring a respectful and informed approach to understanding the complexities of stigma and disability in Sierra Leone.

## Results

In this section we will first describe the sample demographics (Table 2), followed by the results for each of the three research questions with illustrative quotes. An overview of the coding scheme can be found in Table 3. Krio is a distinct language with its own grammar and structure, though it has roots in English. In translating participant quotes, we have aimed to preserve their original meaning and tone while making minor adjustments for clarity where necessary.

**Table 2:** Demographics of study participants.

Characteristics	*n*	%
Gender of participating caregiver		
Female (F)	31	84
Male (M)	6	16
Gender of child living with disabilities		
Male (M)	25	68
Female (F)	12	32
Caregiver relation to child		
Biological parent	29	78
Adopted parent	3	8
Grandparents	3	8
Other relatives (aunt, uncle)	2	5
Age of the child		
Under 1 year	1	3
1 year	3	8
2–5 years	14	38
6–10 years	16	43
11–13 years	3	8
Disability of the child		
Hemiplegic cerebral palsy	14	38
Quadraplegic cerebral palsy	12	32
Diplegic cerebral palsy	3	8
Acquired brain injury	2	5
Epilepsy	1	3
Spastic cerebral palsy	1	3
Muscular dystrophy	1	3
Unilateral amputee	1	3
TB of the spine	1	3
Missing	1	3

**Table 3:** Coding scheme.

Codes	Subcodes
Experiences of stigma by caregivers (RQ1)	Abusive language and derogatory remarks Labeling Avoidance and exclusion Rejection by family Enforced conformity to traditional practices
Caregivers responses to experiences of stigma by association (RQ2)	Withdrawing Shame Hiding Emotional strain Coping Focusing on positive interactions Spiritual reframing Maintaining hope in a cure Ignoring Resisting Challenging superstitious beliefs and practices Showing the child in public Expressing public anger
Caregivers’ experience and wishes for social support (RQ3)	Empathy and awareness Practical support for daily needs Social inclusion Encouragement

### Sample demographics

A total of 37 caregivers of CLWDs participated in our study (see Table 2 for participant demographics). The sample consisted predominantly of women (84%). Among the participants, 29 (78%) were the biological parents of a child with a disability, 3 (8%) were grandparents, 3 (8%) were adoptive parents and in 2 cases (5%), other relatives participated. The CLWDs looked after by our study participants varied in age, with the majority between 2 and 10 years (30 children, 81%) as well as different types of disabilities, including hemiplegic cerebral palsy (38%), quadriplegic cerebral palsy (32%) and acquired brain injury (5%).

### Experiences of stigma by association among caregivers of CLWDs (RQ1)

Our analysis revealed five key social manifestations of stigmatization caregivers experience: *abusive language and derogatory remarks****,**** labeling, avoidance and exclusion****,**** rejection by family****,*** and *enforced conformity to traditional practices****.*** These categories highlight the societal responses to caregivers and their children, as described by the participants in our study.

*Abusive language and derogatory remarks*. Participants in our study described instances where they were subjected to derogatory remarks targeting both their children and themselves. Such remarks often arose during conflicts or everyday interactions and reflected negative societal attitudes toward CLWDs and their caregivers:

Like for me, when you and your neighbor have little argument, they will find a word to tell you. Any small thing that will happen they will say, ‘just leave us alone, look at the demonic child you are having, look at the cripple, she is a devil child who does not walk.’ (Group 1, Participant 2)

According to our sample, abusive language and public shaming remarks were uttered in both private and public encounters, contributing to a sense of social hostility and reinforcing stigmatization.

*Labeling.* Participants described how labeling was used as a social mechanism to identify both them and their children, often reducing their identities to the child’s disability. Unlike overt abusive language or derogatory remarks, labeling was described as a more pervasive, subtle, and socially normalized behavior. It involved the use of the child’s disability as a defining characteristic for both the child and the caregiver, reinforcing their ‘otherness’ within the community. This practice frequently emerged in daily conversations, where individuals would refer to the caregiver or child by the disability rather than their names or personal qualities:

When people refer to us, they will say things like, ‘Kadija’s child, the one with the amputated hand.’ People attach the child’s condition to their identity – and to the caregiver too. For me, they say, ‘That woman with the child who cannot talk.’ These are the challenges we face every day. (Group 3, Participant 5)

*Avoidance and exclusion.* Some participants reported that they and their children were avoided by others in their communities. This avoidance was particularly pronounced among caregivers with children with congenital or early-onset conditions, like cerebral palsy, which are culturally linked to supernatural causes. Participants described how these behaviors reduced opportunities for social interaction and placed a strain on relationships within their communities:

It is not easy for me. Some people won’t even let their children play with my other children because they believe I have given birth to devils or demons. But I know that it is the Almighty God who gave me these children. (Group 4, Participant 1)

Participants also reported being excluded from social places, including being forced to leave their communities due to the stigma associated with their children’s conditions:

Due to the condition of my child, I have been given a notice to quit from my place. (Group 3, Participant 1)

*Rejection by family* was a repeatedly mentioned form of stigma participants described. Reported family responses varied from indifference to rejection by close and extended family members. This lack of familial support was described as a source of additional challenges in managing caregiving responsibilities. For example:

Like for my own, the dad had passed away in 2013 and my husband’s family does not want to know anything [about us]. When the child had a problem, I told all of them and no one wanted to know about us, they do not care. I told them about this operation but still they do not care and left me with nothing. (Group 1, Participant 2)

*Enforced conformity to traditional practices*. Participants in our study described instances where societal and cultural beliefs about disability led to coercion into traditional rituals or practices. These expectations were often rooted in perceptions of disability as a curse or a sign of spiritual imbalance. Caregivers reported being pressured to comply with these beliefs, sometimes against their own judgment or will. One participant recounted their experience:

My husband’s family said we should go to the bush. We went with her to traditional healers and they said for me to return her, like traditionally she is a devil, and they have a way to make her return to the bush by doing some traditional rites. (Group 2, Participant 2)

According to the participant, the pressure to perform these rites reflects the societal demand to align with traditional explanations and solutions for disability, which tends to place responsibility on the caregiver to address the perceived spiritual cause.

#### Summary of RQ 1

Caregivers of CLWDs in our study described various manifestations of stigma they experience in their everyday lives, where societal attitudes and cultural beliefs about disability stigmatized not only the child but also the caregiver. These included abusive language, labeling, avoidance and exclusion, rejection by family, and enforced conformity to traditional practices, highlighting the intersecting stigma faced by both child and caregiver. While the majority of participants reported experiencing stigma in various forms, only one participant reportedly had not encountered stigmatizing attitudes or behaviors against themselves or the child.

### Caregivers responses to experiences of stigma (RQ2)

This section presents the findings on caregivers’ responses to stigmatization, categorized into three categories: withdrawing, coping, and resisting. Each category includes several subcategories that capture the specific strategies caregivers employed to navigate the stigma they and their child experience.

#### Withdrawing

The first category identified in caregivers’ responses to stigma is withdrawing, which encompasses actions and emotions aimed at minimizing exposure to social judgment and ridicule. Subcategories of withdrawing include *shame*, *hiding,* and *emotional strain*.

*Shame* emerged as an internalized emotional response to societal judgment, shaping how caregivers of CLWDs navigated their daily lives. Participants described shame as stemming from societal expectations and the perception of their child’s disability as a mark of personal or familial inadequacy. According to some participants, this internalized stigma led to a reluctance to be seen in public with their child or to engage in community activities. The fear of judgment—whether expressed through looks, whispers, or outright comments—profoundly influenced caregivers’ actions and decisions. As one participant recounted:

Sometimes I feel very ashamed walking with her or even taking her to physiotherapy, unless my relatives encourage me to go. For a young woman like me, facing these challenges is not easy. I cry at home. (Group 2, Participant 5)

*Hiding* reflected a behavioral response to the feelings of shame and fear of societal judgment. Some caregivers reported actively avoiding public spaces or situations where their child’s disability might be noticed, choosing instead to keep their child at home. This action was described as a protective mechanism to shield both the caregiver and the child from ridicule or negative attention. By limiting their public presence, caregivers sought to reduce potential exposure to stigma. One participant shared:

If I am in church and they ask me to walk with my child, he will suddenly shout and stretch, and then people start noticing his abnormality. They begin to see that he is not like other children. That is why I feel ashamed. Right now, I don’t take him out … I keep him at home for his medicine and therapy. (Group 2, Participant 4)

This account underscores how hiding served as a response to stigma, offering temporary relief from external judgment but at the cost of increased isolation and limited social interaction.

*Emotional strain*. Caregivers also described experiencing emotional strain as a result of stigma. This strain, distinct from shame, encompassed the physical and mental toll of worry and helplessness about their child’s condition and the associated stigma. Participants noted that this distress impacted both their mental health and physical well-being, sometimes interfering with their ability to care for their child:

My wife cannot eat, she has lost her appetite due to the condition of our child, and she is seriously losing weight. (Group 5, Participant 3)

#### Coping

The second category identified in caregivers’ responses to stigma is coping, which refers to strategies caregivers employed to navigate the challenges of stigma while maintaining their emotional well-being and caregiving responsibilities. Subcategories of coping include *focusing on positive interactions, spiritual reframing, maintaining hope in a cure,* and *ignoring*.

*Focusing on positive interactions*. Several caregivers described finding moments of joy in their daily lives with their children as a way to cope with stigma. These moments often involved engaging in playful or affectionate activities, which helped strengthen the bond between the caregiver and child and created a sense of positivity. One participant recounted how laughter became an important part of their interactions:

Usually when I used to wash him and crack jokes with him, playing with his jaw, he will burst into laughter. Some will even ask, ‘Is that child the one laughing so loudly?’ People used to ask whenever I play with him if it is really him who laughs so loudly. (Group 4, Participant 7)

In several FGDs, caregivers reported about such interactions, providing them with a sense of fulfillment and reinforcing their commitment to caring for their child in the face of societal stigma.

*Spiritual reframing*. Spiritual beliefs were described by caregivers as a central coping mechanism, allowing them to deal with the stigma associated with their child’s disability. Caregivers described upholding beliefs that their child’s condition was part of God’s plan, suggesting that the child’s existence had a divine purpose. This perspective provided caregivers with a counter-narrative to the stigmatizing views imposed by their communities and attach meaning to their experiences, as the following quotes illustrate:

It is God that gave me her, it is the God that will help me to sustain her. (Group 1, Participant 4)

Additionally, caregivers in our sample emphasized the belief that their child was a divine gift, fostering a sense of acceptance and love. This positive reframing was described as helping them to focus on their child’s inherent worth and reinforced their commitment to caregiving despite societal judgment. One participant described:

We believe when God has given us this kind of person, we should take good care of them and give them the same love we give the others who are not disabled and assure them they are going to be the same. (Group 2, Participant 5)

Despite accepting their child’s conditions, the great majority of our participants expressed *hope for a cure*, attributing this hope to various sources, including divine intervention, medical advancements, or a combination of forces. This hope enabled caregivers to sustain their efforts and focus on the future while managing current challenges.

I have faith that my child will walk and that my child is not a demon. She will get up one day and start to walk. (Group 1, Participant 5)

*Ignoring* negative comments or attitudes from others was described as another coping mechanism. By choosing not to engage with stigmatizing behavior, caregivers attempted to protect their emotional well-being and maintain a sense of control in social situations. Ignoring negative remarks was described to serve as a way to disengage from stigma while maintaining composure and not losing face:

Like when there is a birthday and you say, ‘What about my child?’ They will say, ‘That one is not walking.’ … They look at her as if she is not a good child. So, you think when a person can not walk that means she is not good? These times, I just look at them and ignore. (Group 1, Participant 1)

#### Resisting

The third category identified in caregivers’ responses is resisting, which captures actions caregivers took to challenge stigma and advocate for their children. Subcategories of resisting include *challenging superstitious beliefs and practices, showing the child in public,* and *expressing public anger*.

*Challenging superstitious beliefs and practices*. Caregivers described efforts to counteract harmful beliefs about disability by rejecting superstitions and advocating for a more informed understanding of their child’s condition. One participant shared how they refused to conform to traditional healing practices:

In my area, some people are saying it’s witches or maybe one family member who hates us has done this to us, or they have pushed him to witchcraft. [They say] that I should go to a traditional healer. I said no, this is what God gave me. I will not go to a traditional healer. (Group 1, Participant 6)

In addition to rejecting superstitions, some caregivers educated others about the nature of their child’s condition. By explaining the medical basis for disability, they sought to challenge misconceptions and reduce stigma. One participant recounted:

They [neighbors] normally said she is a devil child, do not go close to her, do not go near my child so the demon does not transfer. … I do tell people it does not transfer because if it was that way, I should have gotten it a long time ago or her elder sister should have contracted it. … Now I am seeing that they encourage her, she is going close to them, and they sit and talk with her. (Group 1, Participant 5)

*Showing the child in public*. Various participants also described taking their child into public spaces as a way of challenging societal misconceptions and asserting their child’s rightful place in the community. By doing so, they hoped to inspire acceptance and demonstrate pride in their child. One participant explained:

I believe that the more we have the courage to take our children out, the more the community will have courage and will help them and accept the situation as it is. After all, it is a decision from God, and if you have courage to not hide your child, it is in that situation that God will bring a solution for you. (Group 6, Participant 5)

This act of visibility served as a form of advocacy, encouraging others to reconsider their attitudes toward CLWDs.

*Expressing public anger*. Two participants also described instances where they confronted individuals in their communities who perpetuated stigma. By expressing anger publicly, they aimed to resist discrimination and create an environment where the child and caregiver could live without fear of ridicule. A mother of CLWD explained:

I used to talk back to people, I sometimes got into serious confrontations between me and people in the community who spoke badly about my child. … It got better, and no one from my community speaks badly about my child to me anymore. (Group 5, Participant 2)

#### Summary for RQ 2

In response to the experiences of stigma, caregivers of CLWDs in our study employed three overarching types of responses: withdrawing, coping, and resisting. *Withdrawing* primarily involved actions and emotions aimed at minimizing exposure to societal judgment. The coded extracts from the data often reflected an inward focus, where caregivers sought to protect themselves and their children by retreating from public spaces or interactions. *Coping*, in contrast, was characterized by adaptive strategies aimed at maintaining emotional well-being and caregiving responsibilities. These responses tended to reflect resilience, demonstrating caregivers’ efforts to find meaning and positivity despite the stigma they faced. *Resisting* represented a more proactive and outward-facing response, involving direct actions to challenge stigma, reshaping societal attitudes and asserting their child’s place within the community. Across these categories, *coping* and its subcategories were mentioned most frequently by participants, suggesting that many caregivers leaned on adaptive strategies as their primary mode of managing stigma. However, participants’ responses also appeared to vary based on the specific situations they encountered. For instance, withdrawing might have been more common in moments of heightened societal judgment, while resisting was employed when caregivers felt compelled to defend or advocate for their child.

### Caregivers’ experience and wishes for social support (RQ3)

This section presents the findings on caregivers’ experiences and wishes for social support, categorized into *empathy and awareness, practical support for daily needs, social inclusion,* and *encouragement*.

*Empathy and awareness*. Participants emphasized the need for empathy and a deeper understanding of their family’s situation from those around them. Caregivers noted that while they encountered moments of sympathy and recognition, many expressed a desire for greater awareness within their communities to reduce stigma and foster acceptance. One participant shared how helpful it was to her, knowing that those around her accepted her child’s disability as an illness:

Like for me, they [community] know it’s a disease and everyone is feeling sorry for me because most of them know it is a disease. No one points a finger at me; they feel sorry for me. They say, ‘This child that was strong, was dancing, now she is not walking anymore,’ and they all know it is a disease. … It’s not easy, but it makes me feel better. (Group 1, Participant 2)

*Practical support for daily needs*. Caregivers described the overwhelming demands of caregiving and their need for practical, hands-on support to ease their daily responsibilities as a form of socially desired support. Many felt that assistance with caregiving tasks, such as childcare or household chores, would also help with the emotional strain they experienced. As one caregiver explained:

The other support that I would need is if someone could offer to take these children from me and help me to take care of them. That is a venture that I will strongly appreciate. (Group 4, Participant 1)

The importance of *social inclusion* was often mentioned in our study, as participants described how invitations to community events provided a sense of belonging for both their children and themselves. Being included in these activities signified that their children were valued members of the community, which fostered acceptance and strengthened social ties.

When there is something in the community for children, they will invite him. There is that concern for him in this new community. I thank God for that. (Group 6, Participant 3)

For birthdays, in school and church, I want my child to be part of this (Group 5, Participant 1)

Such inclusion in social events allowed CLWDs to engage socially and develop relationships as well as reducing the caregivers sense of isolation.

*Encouragement* from the community emerged as another form of support. Participants described how positive reinforcement and advice to remain committed to their caregiving responsibilities motivated them and reaffirmed their efforts. Moreover, some participants expressed that encouragement offered emotional validation and a sense of solidarity among parents as caregivers, as the following quote shows:

In my new community, the people there are very much accommodating to my child. People even used to advise that I should not relent in providing care for my child as I may not know what the future holds for me. … It lifts me up when they say such words. (Group 6, Participant 5)

#### Summary for RQ 3

Caregivers of CLWDs in our study expressed diverse experiences and wishes for social support, encompassing empathy and awareness, practical support for daily needs, social inclusion, and encouragement. They highlighted the importance of community understanding as well as the need for hands-on assistance to ease caregiving burdens. Invitations to community events fostered social inclusion and a sense of belonging, while words of encouragement provided emotional strength and motivation. We found mixed views in our data—most participants had experienced some form of support, but there was an overall wish for more comprehensive and holistic social support that addresses both practical and emotional needs, fostering a more inclusive and understanding community for caregivers and their children.

## Discussion

### Recap of study objectives and key findings

This study aimed to explore the experiences of intersecting forms of stigma among caregivers of children living with disabilities (CLWDs) in Sierra Leone. Using focus group discussions with caregivers, we sought to investigate how overlapping forms of stigma—including stigma by association, blame and culpability ascribed to caregivers, and internalized affiliate stigma—shape their daily lives. We also examined the strategies caregivers employ to navigate these stigmatizing experiences, and the support systems they rely on or desire. Most caregivers in our sample were caring for children with cerebral palsy, a condition acquired prenatally, during birth, or shortly after. Cerebral palsy’s unclear etiology within local frameworks of understanding leaves it particularly vulnerable to supernatural explanations, such as curses, moral failings, divine punishment or bad luck, that could extend to or affect others, and consequently stigmatization of both the child and the caregivers.

Our findings confirmed the multifaceted, pervasive nature of stigma, with entangled layers of stigmatization directed at both the child and caregiver. Caregivers experienced various forms, such as abusive language, labeling, avoidance, and enforced conformity to traditional practices as also found by Yoder and colleagues^65^. While these experiences often emerged in response to the child’s condition, they frequently spilled over onto the caregiver, through proximity (stigma by association), social narratives that attributed them to the child’s conditions, and the internalization of stigmatizing beliefs, or affiliate stigma. In many accounts, these different forms of stigma were so intertwined that they could not be analytically separated. Our study illustrates the relational nature of stigma in Sierra Leone, as it is socially normalized and embedded in everyday conversations^6,18^. This also reflects patterns observed in other contexts where disability becomes compounded by social ideals of ‘normalcy’ that marginalize those who deviate from these norms^17,66^.

Despite the widespread stigma, caregivers in our sample described a variety of responses in dealing with their stigma. Coping strategies were particularly prevalent, with many caregivers relying on spiritual reframing to reinterpret their child’s condition as part of God’s plan or a divine blessing. This aligns with previous research highlighting the role of religious beliefs in mitigating stigma in comparable cultural contexts^10,20^. Notably, many study participants also expressed a deep acceptance of their children, focusing on positive interactions and rejecting societal negativity. This acceptance is noteworthy, given the cultural narratives around disability in which these caregivers were socialized and presumably had internalized such beliefs themselves. It highlights their capacity to adapt and reconcile societal constructs with their lived experiences, even within highly stigmatizing environments^14^. However, it is important to consider that the caregivers in this study were those who actively sought support for their children and were willing to participate in focus group discussions. This may indicate a self-selecting group with greater openness to accepting their child’s condition, a point further discussed in the study’s limitations.

Resistance strategies, though less common than coping, were another noteworthy finding. Caregivers described educating others about their child’s condition, rejecting superstitions, and advocating for inclusion. These acts of resistance challenge societal norms and may reflect a shift toward empowerment and advocacy, despite the societal risks associated with confronting deeply ingrained cultural beliefs. The presence of such strategies in a context where conformity with the collective is valued highlights the caregivers’ agency and the potential for social change.

Social support, or the lack thereof, emerged as a critical theme in caregivers’ experiences within our study, a finding present also in other studies^37,64^. While some caregivers described moments of empathy and inclusion, such as invitations to community events, these instances appeared to be isolated and insufficient. Most caregivers expressed a desire for more holistic and consistent support, including practical assistance with caregiving responsibilities and greater awareness within their communities. This aligns with broader research emphasizing the importance of social inclusion and practical support in mitigating the impacts of stigma^7,67^.

### Theoretical and practical implications

This study contributes to the theoretical understanding of stigma experienced by caregivers of CLWDs by emphasizing the compounded nature of stigma they experience, especially in contexts where cultural narratives link certain disabilities to moral failings or supernatural causes^20,61^. While existing theories often focus on stigma as a relational dynamic between the stigmatized individual and their social environment^16,49,68^, our findings point to the need for frameworks that account for intersecting forms of stigma experienced by caregivers^38^. These include stigma by association, external attributions of blame or culpability for the child’s condition, and the internalization of stigma-related beliefs. Together, these forms converge in ways that shape caregivers’ emotional, social, and moral positioning. Integrating constructs from the social-ecological model could provide a more nuanced understanding of how societal structures, cultural narratives, and individual coping mechanisms interact to shape the lived experiences of caregivers^3,22^. By addressing these intersections, future theories can better account for the compounded effects of stigma in diverse cultural contexts, ultimately improving their relevance and applicability in global disability studies.

This study’s findings underscore the importance of designing caregiver support strategies that are sensitive to both cultural norms and contextual realities. Interventions could prioritize community-level engagement to encourage inclusive narratives around disability, fostering greater empathy and understanding^67,69^. For example, facilitating dialogues that draw on existing cultural frameworks to present disability as a natural or divine variation may help reduce stigma while respecting local beliefs^38^. Practical strategies might include developing caregiver peer networks and counseling that provide emotional support and shared advocacy opportunities, recognizing the importance of solidarity in mitigating stigma. Additionally, community-led initiatives that increase caregivers’ visibility and inclusion—such as social events or collective projects—could help normalize their participation and challenge exclusionary practices^69^. Collaborating with local organizations and stakeholders can ensure that such efforts are grounded in the specific needs and cultural contexts of the communities they serve.

### Limitations and future research

This study provides valuable insights into the compounded experiences of stigma among caregivers of CLWDs in Sierra Leone. However, several limitations should be noted. First, while we strived to approach this research with cultural awareness and empathy, we recognize that, as researchers who have not personally experienced these intersecting stigmas, our interpretations are inevitably shaped by our positionality. Second, our overall relatively small sample predominantly consisted of female caregivers (84%), reflecting caregiving norms in Sierra Leone, but limiting exploration of gendered differences in stigma experiences. Future research could investigate how male caregivers experience and respond to stigma. Third, a potential selection bias may have influenced the findings, as most participants expressed positive attitudes toward their children. Caregivers struggling with internalized stigma or negative beliefs, such as viewing the child’s condition as a curse, may have been underrepresented. Fourth, this study was conducted in Freetown, the capital city, and does not capture the experiences of caregivers in rural or remote areas, where access to education and healthcare is more limited and traditional beliefs about disability may be even more deeply ingrained.

Fifth, for ethical reasons, we did not directly ask caregivers whether they felt personally responsible for their child’s condition, nor did we probe deeply into the spiritual or cultural beliefs they may have internalized in the context of a group setting. While some participants reported being blamed for their child’s disability, their own sense of culpability remains unexplored. Future studies could sensitively examine the dynamics of dual stigma, including internalized guilt. Additionally, the experiences of CLWDs themselves were not included, leaving a gap in understanding how children perceive stigma and their caregivers’ responses. Finally, this study captured experiences at a single point in time, leaving questions about how stigma and coping strategies evolve over the long term. Research on protective factors, such as community characteristics or access to formal support systems, could also shed light on why some caregivers report less stigma. Evaluating community-based interventions to reduce stigma and support caregivers would be another valuable area for future research.

## Conclusion

This focus group study explored the intersecting forms of stigma experienced by caregivers of CLWDs in Sierra Leone. Caregivers detailed experiences of labeling, avoidance, and societal blame rooted in cultural and spiritual beliefs about disability, revealing the profound social challenges they face. These experiences encompassed stigma by association, external attributions of culpability, and internalized stigma, which appeared to overlap. Despite these obstacles, caregivers demonstrated resilience and agency, employing strategies to navigate stigma, including coping through spiritual reframing and, in some cases, resisting societal prejudices. The findings underscore the need for greater understanding and support for caregivers, including interventions that address practical needs and foster social inclusion. By amplifying the voices of caregivers, this study contributes to the broader discourse on stigma, disability, and caregiving, offering insights to inform inclusive policies and culturally sensitive interventions.

## Electronic supplementary material

Below is the link to the electronic supplementary material.

Supplementary Material 1

## Data Availability

The datasets used during the current study are available from the corresponding author on reasonable request.

## Abbreviations

CLWD: Children living with disabilities
ETC: Enable the children
FGD: Focus group discussion
NGO: Non-governmental organization
PLWD: People living with disabilities
RQ: Research question

## References

[1] Goffman, E. *Stigma: Notes on the Management of Spoiled Identity* (Simon & Schuster, 1963).

[2] Link, B. & Hatzenbuehler, M. L. Stigma as an unrecognized determinant of population health: Research and policy implications. *J. Health Polit. Policy Law* **41**, 653–673. 10.1215/03616878-3620869 (2016). 27127258 10.1215/03616878-3620869

[3] Scambler, G. Heaping blame on shame: ‘Weaponising stigma’ for neoliberal times. *Sociol. Rev.* **66**, 766–782. 10.1177/0038026118778177 (2018).

[4] Berry, C. & Greenwood, K. Direct and indirect associations between dysfunctional attitudes, self-stigma, hopefulness and social inclusion in young people experiencing psychosis. *Schizophr. Res.* **193**, 197–203. 10.1016/j.schres.2017.06.037 (2018). 28693753 10.1016/j.schres.2017.06.037

[5] Curra, J. *The Relativity of Deviance* (Sage, 2020).

[6] Andregård, E. & Magnusson, L. Experiences of attitudes in Sierra Leone from the perspective of people with poliomyelitis and amputations using orthotics and prosthetics. *Disabil. Rehabil.* **39**, 2619–2625. 10.1080/09638288.2016.1236409 (2017). 27829289 10.1080/09638288.2016.1236409

[7] Addison, M. Framing stigma as an avoidable social harm that widens inequality. *Sociol. Rev.* **71**, 296–314. 10.1177/00380261221150080 (2023). 37333761 10.1177/00380261221150080 PMC7614666

[8] Albrecht, G. L., Walker, V. G. & Levy, J. A. Social distance from the stigmatized. A test of two theories. *Soc. Sci. Med.* **16**, 1319–1327. 10.1016/0277-9536(82)90027-2 (1982). 6214849 10.1016/0277-9536(82)90027-2

[9] Watermeyer, B. & Swartz, L. Disablism, identity and self: Discrimination as a traumatic assault on subjectivity. *Commun. Appl. Soc. Psychol.* **26**, 268–276. 10.1002/casp.2266 (2016).

[10] Frobisher, E., Elbers, W. & Okwany, A. Gendered disability advocacy: Lessons from the girl power programme in Sierra Leone. In *The Routledge Handbook of Disability Activism* 2021st edn (eds Berghs, M. et al.) 412–427 (Routledge, 2019).

[11] Lalvani, P. Disability, stigma and otherness: Perspectives of parents and teachers. *Int. J. Disabil. Dev. Educ.* **62**, 379–393. 10.1080/1034912X.2015.1029877 (2015).

[12] Scavarda, A. The shame–blame complex of parents with cognitively disabled children in Italy. *Sociol. Health Illn.* **46**, 966–983. 10.1111/1467-9566.13742 (2024). 38165697 10.1111/1467-9566.13742

[13] Mak, W. W. S. & Cheung, R. Y. M. Affiliate stigma among caregivers of people with intellectual disability or mental illness. *Res. Intellect. Disabil.* **21**, 532–545. 10.1111/J.1468-3148.2008.00426.X (2008).

[14] May-Machunda, P. M. Living in the nexus of disability and caregiving: An African American parental caregiver’s critical observations as a folklorist. *J. Am. Folk.* **137**, 354–366. 10.5406/15351882.137.545.08 (2024).

[15] Pryor, J. B., Reeder, G. D. & Monroe, A. E. The infection of bad company: Stigma by association. *J. Pers. Soc. Psychol.* **102**, 224–241. 10.1037/a0026270 (2012). 22082057 10.1037/a0026270

[16] Birenbaum, A. On managing a courtesy stigma. *J. Health Soc. Behav.* **11**, 196. 10.2307/2948301 (1970).

[17] Bayat, M. The stories of ‘snake children’: Killing and abuse of children with developmental disabilities in West Africa. *J. Intellect. Disabil. Res.* **59**, 1–10. 10.1111/jir.12118 (2015). 24467696 10.1111/jir.12118

[18] Berghs, M. & Dos Santos-Zingale, M. A comparative analysis: Everyday experiences of disability in Sierra Leone. *Afr. Today* **58**, 18–40 (2011).

[19] Etieyibo, E. Rights of persons with disabilities in Nigeria. *AF*10.21825/af.v33i1.16559 (2020).

[20] Tilahun, D. et al. Stigma, explanatory models and unmet needs of caregivers of children with developmental disorders in a low-income African country: A cross-sectional facility-based survey. *BMC Health Serv. Res.* **16**, 152. 10.1186/s12913-016-1383-9 (2016). 27117326 10.1186/s12913-016-1383-9 PMC4847244

[21] Link, B. G., Phelan, J. C. & Hatzenbuehler, M. L. Stigma as a fundamental cause of health inequality. In *The Oxford Handbook of Stigma, Discrimination, and Health* (eds Major, B. et al.) 53–68 (Oxford University Press, 2018). 10.1093/oxfordhb/9780190243470.013.4.

[22] Link, B. G. & Phelan, J. C. Conceptualizing stigma. *Annu. Rev. Sociol.* **27**, 363–385. 10.1146/annurev.soc.27.1.363 (2001).

[23] Major, B. et al. (eds) *The Oxford Handbook of Stigma, Discrimination, and Health* (Oxford University Press, 2018).

[24] Tyler, I. *Stigma* (Zed Books Ltd, 2020).

[25] Kassah, A. K. Begging as work: A study of people with mobility difficulties in Accra, Ghana. *Disabil. Soc.* **23**, 163–170. 10.1080/09687590701841208 (2008).

[26] Oliver, M. *The Politics of Disablement* (Macmillan Education UK, 1990). 10.1007/978-1-349-20895-1.

[27] Shakespeare, T. *Disability Rights and Wrongs Revisited* (Routledge, 2013).

[28] World Health Organization. *World Bank* (WHO, 2011).

[29] World Health Organization and the United Nations Children’s Fund (UNICEF). *Global Report on Children with Developmental Disabilities: From the Margins to the Mainstream* (WHO and UNICEF, 2023).

[30] Green, S. E. “What do you mean ‘what’s wrong with her?’”: Stigma and the lives of families of children with disabilities. *Soc. Sci. Med.* **57**, 1361–1374. 10.1016/s0277-9536(02)00511-7 (2003). 12927467 10.1016/s0277-9536(02)00511-7

[31] Wood, T. Subversion of the violent gaze. In *Beauty, Violence, Representation* 1st edn (ed. Dickson, L.) (Routledge, 2014).

[32] Calder-Dawe, O., Witten, K. & Carroll, P. Being the body in question: Young people’s accounts of everyday ableism, visibility and disability. *Disabil. Soc.* **35**, 132–155. 10.1080/09687599.2019.1621742 (2020).

[33] Barbareschi, G., Carew, M. T., Johnson, E. A., Kopi, N. & Holloway, C. “When they see a wheelchair, they’ve not even seen me”—Factors shaping the experience of disability stigma and discrimination in Kenya. *Int. J. Environ. Res. Public Health*10.3390/ijerph18084272 (2021). 33920601 10.3390/ijerph18084272 PMC8073617

[34] van Olphen, J., Eliason, M. J., Freudenberg, N. & Barnes, M. Nowhere to go: How stigma limits the options of female drug users after release from jail. *Subst. Abuse Treat. Prev. Policy* **4**, 10. 10.1186/1747-597X-4-10 (2009). 19426474 10.1186/1747-597X-4-10 PMC2685368

[35] Chandras, J. (Out)Caste language ideologies: Intersectional raciolinguistic stigma and assimilation from denotified tribal students’ perspectives in rural India. *J. Linguist. Anthropol.* **33**, 161–183. 10.1111/jola.12402 (2023).

[36] Malu, B., Kareepadath Rajan, S., Jindal, N. & Thakur, A. Discrimination experiences of old settlers in Sikkim: A qualitative exploration. *Psychol. Stud.* **69**, 417–431. 10.1007/s12646-023-00743-5 (2024).

[37] Jerwanska, V., Kebbie, I. & Magnusson, L. Coordination of health and rehabilitation services for person with disabilities in Sierra Leone—A stakeholders’ perspective. *Disabil. Rehabil.* **45**, 1796–1804. 10.1080/09638288.2022.2074551 (2023). 35603804 10.1080/09638288.2022.2074551

[38] Mostert, M. P. & Weich, M. M. Albinism in Africa: A proposed conceptual framework to understand and effectively address a continental crisis. *ADRY*10.29053/2413-7138/2017/v5n1a6 (2021).

[39] Mousavi, S. B., Lecic-Tosevski, D., Khalili, H. & Mousavi, S. Z. To be able, or disable, that is the question: A critical discussion on how language affects the stigma and self-determination in people with parability. *Int. J. Soc. Psychiatry* **66**, 424–430. 10.1177/0020764020913308 (2020). 32281903 10.1177/0020764020913308

[40] Ślebodia, A. Stigmatized by the language—Linguistic labels of the disabled peoplein Poland and the United States. *JECS* **4**, 404–412. 10.15503/jecs20132.404.412 (2013).

[41] Agbenyega, J. S. The power of labeling discourse in the construction of disability in Ghana. *Educ. Res. Risks Dilemas* **16**, 1–12 (2003).

[42] Ritsher, J. B. & Phelan, J. C. Internalized stigma predicts erosion of morale among psychiatric outpatients. *Psychiatry Res.* **129**, 257–265. 10.1016/j.psychres.2004.08.003 (2004). 15661319 10.1016/j.psychres.2004.08.003

[43] Kowalski, R. M. & Peipert, A. Public- and self-stigma attached to physical versus psychological disabilities. *Stigma Health* **4**, 136–142. 10.1037/sah0000123 (2019).

[44] Pyszkowska, A. & Stojek, M. M. Early maladaptive schemas and self-stigma in people with physical disabilities: The role of self-compassion and psychological flexibility. *Int. J. Environ. Res. Public Health*10.3390/ijerph191710854 (2022). 36078568 10.3390/ijerph191710854 PMC9518149

[45] Hatzenbuehler, M. L. Structural stigma and the health of lesbian, gay, and bisexual populations. *Curr. Dir. Psychol. Sci.* **23**, 127–132. 10.1177/0963721414523775 (2014).

[46] Ocran, J. “There is something like a barrier”: Disability stigma, structural discrimination and middle-class persons with disability in Ghana. *Cogent Soc. Sci.*10.1080/23311886.2022.2084893 (2022).

[47] Hatzenbuehler, M. L. & Link, B. G. Introduction to the special issue on structural stigma and health. *Soc. Sci Med.* **103**, 1–6. 10.1016/j.socscimed.2013.12.017 (2014). 24445152 10.1016/j.socscimed.2013.12.017

[48] Jackson-Best, F. & Edwards, N. Stigma and intersectionality: A systematic review of systematic reviews across HIV/AIDS, mental illness, and physical disability. *BMC Public Health* **18**, 919. 10.1186/s12889-018-5861-3 (2018). 30049270 10.1186/s12889-018-5861-3 PMC6062983

[49] Kulik, C. T., Bainbridge, H. T. J. & Cregan, C. Known by the company we keep: Stigma-by-association effects in the workplace. *AMR* **33**, 216–230. 10.5465/amr.2008.27752765 (2008).

[50] Felix, E. Marked by association(s): A social network approach to investigating mental health-related associative stigma. *J. Health Soc. Behav.* **66**, 259–275. 10.1177/00221465241261711 (2024). 39081161 10.1177/00221465241261711

[51] van der Sanden, R. L. M., Stutterheim, S. E., Pryor, J. B., Kok, G. & Bos, A. E. R. Coping with stigma by association and family burden among family members of people with mental illness. *J. Nerv. Ment. Dis.* **202**, 710–717. 10.1097/NMD.0000000000000189 (2014). 25198703 10.1097/NMD.0000000000000189

[52] Farrugia, D. Exploring stigma: Medical knowledge and the stigmatisation of parents of children diagnosed with autism spectrum disorder. *Sociol. Health Illn.* **31**, 1011–1027. 10.1111/j.1467-9566.2009.01174.x (2009). 19659737 10.1111/j.1467-9566.2009.01174.x

[53] Francis, A. Stigma in an era of medicalisation and anxious parenting: How proximity and culpability shape middle-class parents’ experiences of disgrace. *Sociol. Health Illn.* **34**, 927–942. 10.1111/j.1467-9566.2011.01445.x (2012). 22280545 10.1111/j.1467-9566.2011.01445.x

[54] Gray, D. E. Ten years on: A longitudinal study of families of children with autism. *J. Intell. Dev. Disabil.* **27**, 215–222 (2002).

[55] Manago, B., Davis, J. L. & Goar, C. Discourse in action: Parents’ use of medical and social models to resist disability stigma. *Soc. Sci. Med.* **184**, 169–177. 10.1016/j.socscimed.2017.05.015 (2017). 28550803 10.1016/j.socscimed.2017.05.015

[56] Song, J., Mailick, M. R. & Greenberg, J. S. Health of parents of individuals with developmental disorders or mental health problems: Impacts of stigma. *Soc. Sci. Med.* **217**, 152–158. 10.1016/j.socscimed.2018.09.044 (2018). 30333078 10.1016/j.socscimed.2018.09.044 PMC6202233

[57] Patel, V. et al. The healthy activity program (HAP), a lay counsellor-delivered brief psychological treatment for severe depression, in primary care in India: A randomised controlled trial. *Lancet* **389**, 176–185. 10.1016/S0140-6736(16)31589-6 (2017). 27988143 10.1016/S0140-6736(16)31589-6 PMC5236064

[58] UNPRPD & UN Sierra Leone. *Situational analysis of the rights of persons with disabilities in Sierra Leone*. Country Brief https://unprpd.org/new/wp-content/uploads/2023/12/Situation_Analysis_CountryBrief_SierraLeone-ac7.pdf. Accessed 31 Jan 2025 (2022).

[59] Srikanthan, S. Contested disability: Sickle cell disease. *Health Soc. Work* **48**, 209–216. 10.1093/hsw/hlad014 (2023). 37315205 10.1093/hsw/hlad014

[60] Olusanya, B. O. et al. Developmental disabilities among children younger than 5 years in 195 countries and territories, 1990–2016: A systematic analysis for the global burden of disease study 2016. *Lancet Glob. Health* **6**, e1100–e1121. 10.1016/S2214-109X(18)30309-7 (2018). 30172774 10.1016/S2214-109X(18)30309-7 PMC6139259

[61] Enoch, A. et al. “We are not getting jobs”: Job seeking problems of people with disability and coping strategies adopted in an urban traditional community in Ghana. *DCID* **27**, 109. 10.5463/dcid.v27i1.499 (2020).

[62] Berghs, M. et al. “You have to find a caring man, like your father!” Gendering sickle cell and refashioning women’s moral boundaries in Sierra Leone. *Soc. Sci. Med.* **259**, 113148. 10.1016/j.socscimed.2020.113148 (2020). 32623231 10.1016/j.socscimed.2020.113148 PMC7322450

[63] Berghs, M. et al. (eds) *The Routledge Handbook of Disability Activism* 2021st edn. (Routledge, 2019).

[64] Morin, K. L. et al. Perspectives on disabilities in Sierra Leone. *J. Spec. Educ.* **56**, 146–157. 10.1177/00224669211065051 (2022).

[65] Yoder, H. N. C., Tol, W. A., Reis, R. & de Jong, J. T. V. M. Child mental health in Sierra Leone: A survey and exploratory qualitative study. *Int. J. Ment. Health Syst.* **10**, 48. 10.1186/s13033-016-0080-8 (2016). 27354854 10.1186/s13033-016-0080-8 PMC4924306

[66] Stone-MacDonald, A. & Digman Butera, G. Cultural beliefs and attitudes about disability in East Africa. *Rev. Disabil. Stud.* **8**, 1–19 (2014).

[67] Hartog, K. et al. Stigma reduction interventions for children and adolescents in low- and middle-income countries: Systematic review of intervention strategies. *Soc. Sci. Med.* **246**, 112749. 10.1016/j.socscimed.2019.112749 (2020). 31978636 10.1016/j.socscimed.2019.112749 PMC7313083

[68] Bos, A. E. R., Pryor, J. B., Reeder, G. D. & Stutterheim, S. E. Stigma: Advances in theory and research. *Basic Appl. Soc. Psychol.* **35**, 1–9. 10.1080/01973533.2012.746147 (2013).

[69] Hearst, M. O. et al. Community-based intervention to reduce stigma for children with disabilities in Lusaka, Zambia: A pilot. *Disabil. Rehabil.* **44**, 2295–2304. 10.1080/09638288.2020.1829105 (2022). 33053312 10.1080/09638288.2020.1829105

