# Supplementary material for: Experiences of stigma among caregivers of children with disabilities in Freetown
Source: Sci Rep. 2025 Jun 20;15:20236. doi: 10.1038/s41598-025-07034-1 (PMC12181342; doi:10.1038/s41598-025-07034-1)
Supplement: Supplementary file 1 — Supplementary Material 1 [file 41598_2025_7034_MOESM1_ESM.pdf]

Semi-structured Interview Guide

Introduction & Consent

Introduction:

- Thank the participants for their time and explain the purpose of the study.
- Emphasize confidentiality and voluntary participation.
- Ask for verbal or written consent.

Warm-up Questions

- Can you tell us a little about yourself and your role as a caregiver?
- How long have you been caring for your child?
- What is your child's age and condition? *(If comfortable sharing.)*

Section 1: Experiences of Stigma by Association

General Perceptions of Disability & Stigma:

- How do people in your community view children with disabilities?
- Have you ever noticed different attitudes toward children with disabilities compared to other children?

Personal Experiences of Stigma:

- Have you ever been treated differently because you are a caregiver of a child with a disability?
  - Have people ever made negative comments about you or your child? What kind of things do they say?
- Can you describe specific situations where you felt stigmatized or judged by others?
- How do people in your family or community talk about your child's condition?
  - How has your family responded to your child's disability? Have you felt supported or abandoned?
  - Have you ever felt excluded from community activities or gatherings because of your child's condition?

Section 2: Caregiver Responses to Stigma

- How does stigma affect you emotionally and mentally?
  - What do you do when you feel judged or excluded?
- Have you ever avoided public places or social events because of the way people treat you or your child?
- What helps you in your daily live to deal cope with your situation?
- How do you react when you get negative comments or reactions? How does that work for you?
- Have you ever confronted people who stigmatized you?
- Have you taken steps to educate others about your child's condition?

Section 3: Social Support & Needs

Existing Social Support:

- Who has been supportive of you in your caregiving journey?

- Have you experienced moments where your community has been understanding or accepting?

Desired Social Support:

- What would you like people in your community to understand better about children with disabilities?
- What kind of help would make your daily caregiving responsibilities easier?
- Have you received encouragement from anyone that has helped you and made you feel supported?

Closing Question

- Is there anything else you would like to share that we haven’t covered?
